# Antibacterial activities of plant leaf extracts against multi-antibiotic resistant *Staphylococcus aureus* associated with skin and soft tissue infections

**DOI:** 10.1186/s12906-022-03527-y

**Published:** 2022-02-21

**Authors:** P. A. Akinduti, V. Emoh-Robinson, H. F. Obamoh-Triumphant, Y. D. Obafemi, T. T. Banjo

**Affiliations:** 1 Microbiology Unit, Department of Biological Sciences, Covenant University, Ota, PMB 1023 Ogun State Nigeria; 2 Department of Microbiology, Crawford University, Igbesa, Ogun State Nigeria

**Keywords:** *S. aureus*, Biofilm, Antibiotic resistance, Plant extracts, Skin and soft tissue infections

## Abstract

**Background:**

The antibacterial activities of aqueous leaf extracts of *Moringa oleifera, Vernonia amygdalina, Azadirachta indica* and *Acalypha wilkesiana* against multidrug resistance (MDR) *Staphylococcus aureus* associated with skin and soft tissue infections were investigated.

**Methods:**

*Staphylococcus aureus* (*n* = 183) from the skin and soft tissue infections with evidence of purulent pus, effusions from aspirates, wounds, and otorrhea were biotyped, and evaluated for biofilm production. The phenotypic antibiotic resistance and MDR strains susceptibility to plant leaves extract were determined using disc diffusion and micro-broth dilution assays respectively. The correlation of plant extract bioactive components with inhibitory activities was determined.

**Results:**

High occurrence rate of *S. aureus* were recorded among infant and adult age groups and 13.2% mild biofilm producers from the wound (*p* < 0.05). Of 60.2% MDR strains with overall significant MARI of more than 0.85 (*p* < 0.05), high resistant rates to linozidine (92.7%; 95% CI:7.27–10.52), ofloxacin (94.2%; 95% CI:6.09–8.15), chloramphenicol (91.2%; 95% CI:6.11–8.32), gentamicin (97.3%; 95% CI:6.20–8.22), ciprofloxacin (92.7%; 95% CI: 5.28–7.99) and vancomycin (86.6%; 95% CI:6.81–9.59) were observed. *Vernonia amygdalina* and *Azadirachta indica* showed significant antimicrobial activity at 100 mg/ml and 75 mg/ml, with low susceptibility of less than 10% to 25 mg/ml, 50 mg/ml, and 75 mg/ml *Moringa oleifera.* Alkaloids, saponin and terpenoids were significant in *Moringa oleifera, Acalypha wilkesiana, Azadirachta indica* and *Vernonia amygdalina* leaves extracts (*p* < 0.05). High inhibitory concentrations at IC50; 3.23, 3.75 and 4.80 mg/ml (*p* = 0.02, CI: − 0.08 – 11.52) and IC90; 12.9, 7.5, and 9.6 mg/ml (*p* = 0.028, CI: 2.72–23.38) were shown by *Acalypha wilkesiana, Vernonia amygdalina and Moringa oleifera* respectively. Comparative outcome of the plant extracts showed *Acalypha wilkesiana*, *Vernonia amygdalina* and *Moringa oleifera* to exhibit significant inhibition activities (*p* < 0.05) compared to other extracts. Significant median inhibitory concentration (15.3 mg/ml) of *Azadirachta indica* were observed (*p* < 0.01) and strong associations of phytochemical compounds of *Azadirachta indica* (eta = 0.527,*p* = 0.017), *Vernonia amygdalina* (eta = 0.123,*p* = 0.032) and *Acalypha wilkesiana* (eta = 0.492,*p* = 0.012) with their respective inhibitory values.

**Conclusion:**

Observed high occurrence rate of skin and soft tissue infections caused by biofilm-producing MDR *S. aureus* requires alternative novel herbal formulations with rich bioactive compounds from *Moringa oleifera, Acalypha wilkesiana, Azadirachta indica* and *Vernonia amygdalina* as skin therapeutic agents.

**Supplementary Information:**

The online version contains supplementary material available at 10.1186/s12906-022-03527-y.

## Background

One of the most common causes of skin and soft tissue infections (SSTIs) is *Staphylococcus aureus* [1] and increases the risk of more invasive systemic infections including bacteremia, septicemia and osteomyelitis [2, 3]. The severity of Staphylococci infection ranges from mild skin abscess, superficial tissue infection to life-threatening diseases [4]. Global epidemiological reports have shown SSTIs are usually aggravated by *Staphylococcus aureus* biofilm, leading to extensive antibiotic resistance, thereby limiting available treatment options [1, 3]. Secretion of extracellular polymeric substance (called biofilm) minimizes therapeutic drug activities and enhances colonization due to polysaccharide intercellular adhesin, a major component of staphylococci biofilm [5, 6]. Formation of Staphylococci biofilm, particularly in wound infection and skin abscess, usually increases severity and chances of bloodstream infection, thereby aiding the SSTIs morbidity mostly among the In-patients [4]. Persistent antibiotic resistance to β-lactams [7], fluoroquinolones [8], and cephalosporins [9], provide a high magnitude of skin morbidity and infection burden.

Due to the poor efficacy of antibiotics against resistant *S. aureus* in SSTIs, selected plant extracts such as *Moringa oleifera, Vernonia amygdalina, Azadirachta indica* and *Acalypha wilkesiana* showed high antimicrobial activities as alternative skin therapy used mainly by numerous infected individuals as local concoction with undocumented successes. These plant extracts are natural, safe, easily accessible, non-toxic with little or no side effects, and with a significant level of phytochemicals expressing higher functional antimicrobial activity against SSTIs than synthetic drugs [10].

Ethnobotanical relevance of leaves parts of *Moringa oleifera* (L) Millsp [11], *Vernonia amygdalina Del.* [12], *Azadirachta indica* Juss [13] and *Acalypha wilkesiana* Mueli. Arg [14] were most used plant parts for medicinal purposes because of their rich and functional bioactive compounds such as anthocyanin, glycosides, coumarins, flavonoids, phenols, saponins, tannins and terpenoids [15]. Use of organic solvents (methanol, ethanol, dichloromethane, and acetone) for plant extraction influenced the antibacterial activities of the extracts due to solvents bacteriostatic or bacteriocidal activities [16, 17], but the aqueous extract of the plant leaves is preferably utilized for the investigation of the anti-staphylococci activities due to high polarity of water as a solvent for plant bioactive compounds and non-inhibitory potential to the organism [18]. To date, anti-staphylococci potential of these plant aqueous extracts from leaves part against SSTIs has not been well explored for scientific evidence. Therefore, the present study investigates the antibacterial activities of leaf extract of *Moringa oleifera, Vernonia amygdalina, Azadirachta indica* and *Acalypha wilkesiana* against multi-resistant *Staphylococcus aureus* associated with skin and soft tissue infections.

## Methods

### Isolate collections

Staphylococci isolates from purulent pus (*n* = 58), effusions from aspirates (*n* = 34), wounds (*n* = 55), and otorrhea (*n* = 36) among out-patients diagnosed with skin and soft tissue infections were selected. Ethical permission from the Health Research Ethics Committees with protocol approval (FMCA/470/HREC/09/2017; NHREC/08/10–2015) was obtained. Each isolate was phenotypically characterized on mannitol salt and Baird-Parker agars and further Gram-stained and biotyped using API as previously described [19].

### Biofilm estimation

The level of biofilm produced from biomass was evaluated in three replicates using standard inoculum of 0.5 McFarland turbidity adjusted to the absorbance of 0.01. Each isolate of 500 μl was distributed in a 96-well plate (Corning, New York, NY, United States) and incubated at 37 °C for 24 h. After incubation, the wells were gently washed three times with phosphate buffer saline (PBS), then 500 μl of 0.2% crystal violet was added to each well and further incubated for 20 min at room temperature. All the wells were washed three times with PBS, and 500 μl of 95% ethanol was added to each well. The intensity of the developed colour was measured at absorbance of 595 nm to evaluate the amount of biofilm biomass, which is proportional to the absorbance value [20]. According to the proportion of biofilm biomass, the biofilm level was classified as strong, mild, and weak as previously described [21].

### Antibiogram

Antibiotic susceptibility pattern of each isolate was determined using disc diffusion method according to Bauer et al. [22] on Mueller–Hinton agar plates. Briefly, a standardized inoculum of 0.5 McFarland turbidity was prepared from pure overnight isolates and tested against antibiotic discs of linozidine (LZD, 30 μg), vancomycin (VA, 30 μg), fosfomycin (FOX, 30 μg), erythromycin (E, 15 μg); trimethoprim/sulfamethoxazole (SXT, 25 μg), ofloxacin (OFX, 10 μg), ceftriaxone (CRO, 30 μg), amoxicillin (AMC, 10 μg), gentamicin (CN, 10 μg), ciprofloxacin (CIP, 10 μg), ceftazidime (CAZ, 30 μg) and tetracycline (TE, 30 μg). Zones of inhibitions were measured after aerobic incubation at 37 °C for 24 h and interpreted according to CLSI guidelines [23]. The multi-antibiotic resistance index (MARI) was determined [19], in which the number of antibiotics an isolate is resistant to is divided by the total number of antibiotics used. Resistance to at least one agent in three or more antimicrobial classes was defined as multidrug-resistant (MDR) and non-MDR as susceptibility to all antibiotics [24].

### Plant collection and authentication

Four plant samples were selected from previous ethnopharmacological studies, based on the frequency of these plant leaves (extracts) for the local treatment of skin infections and the high rate of their citation, as described in Table 1. In addition, these plant leaves are commonly known by their antibacterial efficacy among the locals for treating septic wounds and other skin infections (but not reported in the literature). Fresh leaves of each plant from a single population were collected from the premises of the Covenant University, Ota, Nigeria in February, 2021 with permission from the University Floral management. Taxonomic identification was done by Dr. A. S Oyelakin [25] and deposited with voucher numbers; *Moringa oleifera* (L) Millsp (FHA0025), *Acalypha wilkesiana* Mueli. Arg (FHA007), *Azadirachta indica* Juss (FHA0084) and *Vernonia amygdalina* Del. (FHA0083) at the Federal University of Agriculture, Abeokuta (FUNAAB) Herbarium, Abeokuta, Nigeria.

**Table 1:** Ethnobotanical surveys of selected plants

Plant (Scientific name)	Family	Yielded (g)	Ethnobotanical relevance	Ref
*Vernonia amygdalina* Del.	Asteraceae	7.5	Skin infections and diarrhoea	[12, 26]
*Acalypha wilkesiana* Mueli. Arg	Euphorbiaceae	12.9	Skin infection, antihelmithics, Nosocomial infections	[14, 27]
*Moringa oleifera (L)* Millsp	Moringaceae	9.6	Wound infection, Anti-inflammatory, cytotoxic,	[11, 28, 29]
*Azadirachta indica* Juss	Meliaceae	11.1	Wound therapy, antivirals, anti-inflammatories, Antiseptics, antihelmintics Antibiofilm,	[13, 30, 31]

### Aqueous extraction

Obtained leaves were dried at room temperature, crushed and blended to powdery form. An aqueous extraction was carried out using cold extraction process as previously described [32]. Briefly, powdered leaves were homogenized in separate 500 ml sterile distilled water and maintained at 35 °C for 3 days. Subsequently, the aqueous mixtures were filtered and kept in the oven at 40 °C for 2–3 days. The filtered extracts were concentrated using the rotary evaporator at 70 °C for 2–4 h, and stored at 2–4 °C in sterile bottles.

### Phytochemical analysis

Qualitative phytochemical analysis was carried out to detect the presence of anthocyanin, alkaloid, glycosides, coumarins, flavonoids, phenols, quinons, saponins, tannins and terpenoids in all four extracts following standard procedures described by Chinnadurai et al [33] with slight modifications. Briefly, for anthocyanin detection, 1 ml of 2 N sodium hydroxide (NaOH) was added to 2 ml of filtrates and heated for 5 min at 100 °C in a beaker on a hot plate and 2 ml of Glacial acetic acid (CH_3_COOH) and a few drops of 5% ferric chloride was added to 0.5 ml of the filtered extracts, under the layer of 1 ml of concentrated H_2_SO_4_ for detection of glycosides. The detection of coumarins follows the addition of 1 ml of 10% sodium hydroxide to 1 ml of the extracts and flavonoids was determined by adding 5 ml dilute ammonia solution (NH_3_) to 2 ml of the aqueous filtrates followed by addition of concentrated H_2_SO_4_. Phenol was determined following addition of 2 ml distilled water and few drops of 10% ferric chloride to 1 ml of filtered extracts. Saponin was detected by adding 2 ml sterile distilled water to 2 ml of filtrates, shaken vigorously lengthwise for 5 min and 1 cm formation of foam was observed. Only 2 ml 5% ferric chloride (FeCl_3_) was added to 1 ml of the filtrates and observed for a greenish-black precipitation to detect tannins and 2 ml chloroform was added to 0.5 ml of the filtered extracts for detection of terpenoids.

### Antimicrobial activity of the aqueous extracts

MDR strains among the Staphylococci were selected for the extract susceptibility assay according to the previously described method [34]. Briefly, 0.5 McFarland turbid inoculum was evenly spread on the surface of sterile Mueller–Hinton agar plates, and sterile paper disc previously soaked in a known concentration of extract (20 mg/ml per disc) was gently and firmly placed. Produced inhibition zones were measured after 24 h incubation at 37 °C and compared with the control disc containing only sterile physiological saline. The inhibitory concentration (IC) of each extract was determined using varying dilutions of the aqueous plant extract at concentrations ranging between 0.5 to 64 mg/ml. Serial dilutions of equal volume of 100 μl each of the extract and the nutrient broth was prepared in a sterile microtitre plate and 100 μl of standardized inoculum was added to all the wells. Separate plates for each extract were incubated aerobically at 37 °C for 18–24 h. Test extract control (extract and the growth medium without inoculum) and the organism control (the growth medium, physiological saline and the inoculum) were included. The lowest concentration (the highest dilution) of the extract with no turbidity (no visible bacterial growth) compared with the control tubes were regarded as the inhibitory concentrations.

### Data analysis

The level of significance of the occurrence of suspected *S. aureus* from the clinical samples obtained from various age groups and rates of biofilm production was evaluated with ANOVA at 95 and 99% confidence intervals. To ascertain the differences in rates of the MDR and non-MDR strains, Wilcoxon Signed Rank Test was used to compare at 95% probability level and T-test to estimate the significance of resistance of the antibiotic among the *S. aureus*. Eta-square was calculated as a descriptive measure of the strength of association between phytochemical compounds (independent) and antimicrobial activities of plant extracts (dependent) and further interpreted the Pearson’s coefficient taking the significance at *p* < 0.05. Significance of IC50 and IC90 were evaluated with the phytochemical compound taking the *p*-value < 0.05 at the confident interval of 95%.

## Results

### Prevalence rate of *S. aureus* and degree of biofilm production

**Fig. 1:**
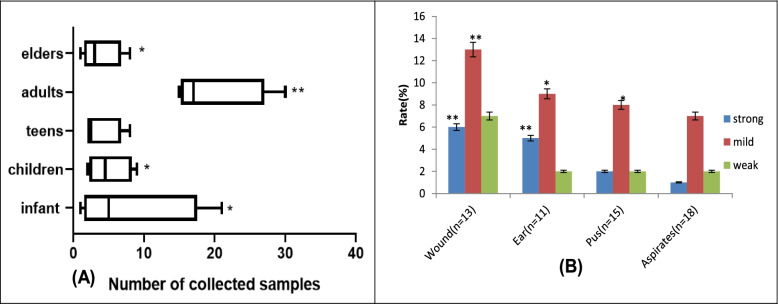
**A** Occurrence rate of suspected *S. aureus* in clinical samples according to the subjects’ age distribution **B** Degree of biofilm production by *S. aureus* in various collected clinical samples (key: **p = 0.01; *p < 0.05)

A significance occurrence rate of suspected *S. aureus* in SSTIs was observed among the infant, children, adult, and elderly age groups (*p* < 0.05) with less number from the teens. The highest median of estimated clinical samples collected was observed among the adult group (Fig. 1A). All the samples harbor *S. aureus* with the potential to produce biofilm regarding the accumulation of the biomass biofilm estimations in all the clinical samples. Significantly high and mild biofilm-producers were observed in wound infections (13.2%) and more than 8.0% rate in ear, pus and aspirates (*p* < 0.05 and *p* = 0.01) (Fig. 1B).

### Multidrug resistance pattern of the *S. aureus* isolates

**Fig. 2:**
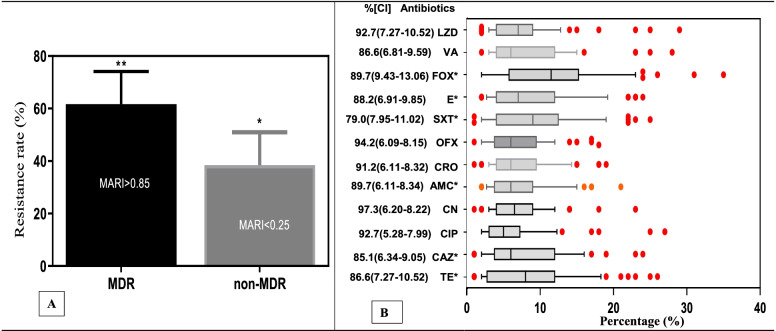
**A** Overall multi-drug resistance (MDR) pattern and multi antibiotic resistance indices (MARI) **B** Box plot evaluation of the antibiotic resistance pattern of *S. aureus* (*p < 0.05; linozidine (LZD); vancomycin (VA); fosfomycin (FOX), erythromycin (E); trimethoprim/sulfamethoxazole (SXT); ofloxacin (OFX); ceftriaxone (CRO); amoxicillin (AMC); gentamicin (CN); ciprofloxacin (CIP); ceftazidime (CAZ), tetracycline (TE))

From the clinical isolates of *S. aureus*, more than 60% were multidrug-resistant strains (showing significant resistance to more than three antibiotic classes) with an overall MARI of more than 0.85 compared to non-MDR strains (*p* < 0.05) (Fig. 2A). The MDR strains showed more than 90% resistant rates to linozidine (92.7%; 95% CI:7.27–10.52), ofloxacin (94.2%; 95% CI:6.09–8.15), chloramphenicol (91.2%; 95% CI:6.11–8.32), gentamicin (97.3%; 95% CI:6.20–8.22), ciprofloxacin (92.7%; 95% CI: 5.28–7.99) and vancomycin (86.6%; 95% CI: 6.81–9.59). Significant median resistance rates of more than 10% were shown to fosfomycin, erythromycin, trimethoprim/sulfamethoxazole, amoxicillin, ceftazidime, tetracycline (*p* < 0.05) (Fig. 2B).

### Susceptibility rates of *S. aureus* to plant extracts

**Fig. 3:**
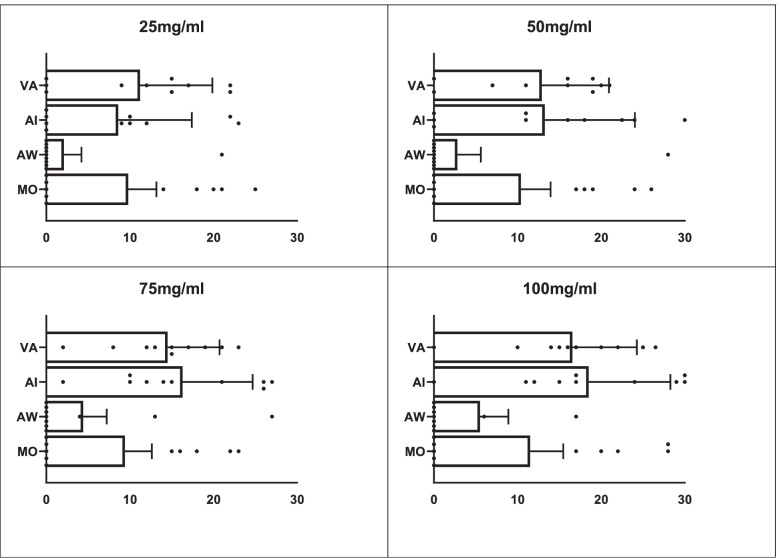
Susceptibility rates of *S. aureus* to *Moringa oleifera (MO), Acalypha wilkesiana (AW), Azadirachta indica* (AI) and *Vernonia amygdalina* (VA) at various dilutions of 100 mg/ml, 75 mg/ml, 50 mg/ml and 25 mg/ml

Susceptibility rates of the *S. aureus* to various dilutions of the plant extracts revealed both *Vernonia amygdalina* and *Azadirachta indica* exhibited significant antimicrobial activity at 100 mg/ml and 75 mg/ml. Low susceptibility of less than 10% to *Moringa oleifera,* was recorded at 25 mg/ml, 50 mg/ml and 75 mg/ml. In contrast, very low susceptibility to *Acalypha wilkesiana* was observed at all the dilutions (Fig. 3).

### Phytochemical composition and antibacterial activities of the plant extracts

The primary estimation of phytochemical composition of the aqueous leave extracts from the plants revealed significant level of alkaloid, flavonoids, phenol, saponin, tannins and terpenoids in *Moringa oleifera, Acalypha wilkesiana, Azadirachta indica* and *Vernonia amygdalina* leaves extracts (*p* < 0.05). Anthocyanin and quinones, glycosides, and coumarins were not detected in *Moringa oleifera*, *Vernonia amygdalina*, and *Acalypha wilkesiana* respectively (Table 2). Significant inhibitory concentrations of 4.8, 3.23, 11.10 and 3.75 mg/ml were shown at IC50 (*p* = 0.02, CI: − 0.08 – 11.52) and 9.6, 12.9, 22.2 and 7.5 mg/ml at IC90 (*p* = 0.028, CI: 2.72–23.38) by *Moringa oleifera, Acalypha wilkesiana, Azadirachta indica* and *Vernonia amygdalina* respectively against the MDR-*S. aureus* (Table 2).

**Table 2:** Phytochemical compounds and antimicrobial activities of plant extracts

Plant extract	Anthocyanin	Alkaloid	Glycosides	Coumarins	Flavonoids	Phenols	Quinons	Saponins	Tannins	Terpenoids	Inhibitory Concentration	
											IC50 (mg/ml)	IC90 (mg/ml)
*Moringa oleifera*	–	++	+	+	++	+	–	+++	+	+++	4.80	9.6
*Acalypha wilkesiana*	++	+	++	–	+	++	+	++	++	++	3.23	12.9
*Azadirachta indica*	+	++	+	++	+++	++	+	++	+	++	11.10	22.2
*Vernonia amygdalina*	+	+++	–	+	++	++	+++	++	++	++	3.75	7.5
p-value	0.391	0.638	0.95	0.95	0.761	0.391	0.058	0.015	0.391	0.031	0.02	0.028
95%CI											−0.08 – 11.52	2.72–23.38

### Comparative evaluation of extracts inhibitory concentration (IC) and association with phytochemical compounds

**Fig. 4:**
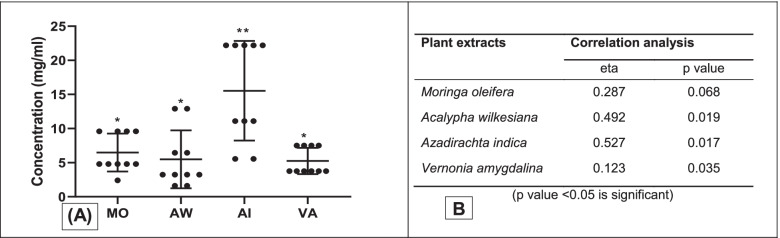
**A** comparative evaluation of the inhibitory concentrations of the plant extracts (*p = 0.05; **p = 0.01) **B** correlation analysis of the phytochemical compounds with the antimicrobial activity

Comparative outcome of the plant extracts showed *Acalypha wilkesiana*, *Vernonia amygdalina* and *Moringa oleifera* to exhibit significant inhibitory activities (*p* < 0.05). Highest and significant median IC (15.3 mg/ml) of *Azadirachta indica* were observed compared to other plant extracts (*p* < 0.01). Significant but low inhibitory concentrations of less than 10 mg/ml were shown by *Moringa oleifera*, *Acalypha wilkesiana* and *Vernonia amygdalina* against the strains (*p* < 0.05) (Fig. 4A). High and significant associations of phytochemical compounds of *Azadirachta indica* (eta = 0.527, *p *= 0.017), *Vernonia amygdalina* (eta = 0.123, *p *= 0.032) and *Acalypha wilkesiana* (eta = 0.492, *p* = 0.012) with their respective IC values were recorded but mild association with *Moringa oleifera* was observed (Fig. 4B).

## Discussion

*Staphylococcus aureus* infection of the furuncles, carbuncles, skin abscesses, and wounds are common skin and soft tissue infections (SSTIs) [3], which usually begin with minor boils and may progress to severe disease conditions associated with subcutaneous tissues, muscle, bones and bloodstream. The study reveals a significant rate of *S. aureus* infectivity in all age groups due to its contagious potential and transmission through skin contact, particularly with animals [19, 35], which is frequently mediated by several adhesins [19]. Biofilm is a major component involved in *S. aureus* invasion. Adherence of *S. aureus* through biofilm, initiates colonization of exposed or broken skin or soft tissue surfaces influenced by the hydrophobic and hydrophilic interactions [36, 37]. Continue spread or dispersal of biofilm encased *S. aureus* in wound or abscess further enhance tissue tropism, extracellular matrix invasion, tissue necrosis and possibly bloodstream infection in cases of severe or immunocompromised conditions [38, 39]. In addition, produced biofilm reduces or prevents the diffusion of antimicrobial drugs, hindering access of the drug to pathogen cells, which is adopted tools to resist antibiotic activity [40, 41].

Evidence of antimicrobial resistance (AMR) among the SSTIs caused by *Staphylococcus aureus* is a major challenge to the treatment and clinical management of abscesses, wound, non-purulent skin and soft tissue infections [42, 43]. Recent reports have shown an increasing prevalence of the SSTIs caused by *S. aureus* across the globe [44, 45]. A high rate of *S. aureus* with MARI of more than 0.85 suggests increasing emergence of community-associated Staphylococci with high MDR pattern to ofloxacin, gentamicin, ciprofloxacin and vancomycin. This further portrays a gradual loss of potency of the available antibiotics for purulent skin and soft tissue infection. This occurrence further suggests the impending emergence of MDR strains, leading to a high spread and pandemic of community-associated SSTIs and debilitating skin morbidity.

The high AMR pattern of *S. aureus* associated with SSTIs presents a worrisome situation that requires urgent and improved strategic intervention*.* There is a need to investigate commonly used indigenous plants leaves for antimicrobial properties as therapeutic options for the treatment of SSTIs. Significant anti-staphylococci activities of low concentrations of *Vernonia amygdalina* and *Azadirachta indica* extracts indicate promising natural antimicrobial products that could provide important opportunities for developing new drug leads for the treatment of SSTIs [46, 47]. Despite low susceptibility to aqueous leaf extracts of *Moringa oleifera and Acalypha wilkesiana,* these extracts produce significant anti-staphylococci activities with inhibition of *S. aureus* isolated from wounds, purulent abscess and severe otorrhea. *Vernonia amygdalina* and *Azadirachta indica* leaves have been reported to be valuable medicinal plant parts with high content of complex active components with functional pharmacological activities showing evident of anti-staphylococci activity [48, 49]. Evidence of significant anti-staphylococci effect of *Vernonia amygdalina, Azadirachta indica*, *Moringa oleifera* and *Acalypha wilkesiana* extracts at concentration of 25 to 100 mg/ml, further emphasizes the ethnopharmacological relevance of these plants as excellent candidates to overcome *S. aureus* infection associated with hospital- and community-acquired skin and soft tissue infections. The significant median inhibitory activity of *Acalypha wilkesiana*, *Vernonia amygdalina* and *Moringa oleifera* to MDR-*S. aureus,* reveal contribution of phytochemical composition of these plant extracts by facilitating the inhibition of staphylococci replication through inactivation of the DNA synthesis [50]. The anti-staphylococci effect could be attributed to expression of alkaloids, saponin, tannin and terpenoids which are reported to cause precipitation of cell wall proteins in Gram positive bacterial, inhibition of microtubules that are essential cytoskeleton, promoting binding to proteins receptors resulting to cell death [51, 52]. The observed inhibitory activities depend on the plant phylogenetic and taxonomic composition that make them suitable alternative anti-staphylococci agents for therapeutic use in cases of SSTIs [15].

Recorded significant associations of phytochemical compounds with respective inhibitory values further prove the anti-staphylococci potential of these plant extracts through the interaction and synergistic activities of detected bioactive compounds. Phenols and flavonoids causes deprivation of metabolized iron or inhibition of hydrogen bonding with vital proteins and microbial enzymes (mostly hyaluranidase and staphylokinase), resulting in complex compounds [53]. According to a previous report, high-level glycosides, which are condensed products of sugars with varieties of organic hydroxyl compounds or its derivatives, could effectively inhibit the efflux pump activity of *S. aureus*, which is one of the potential resistance mechanisms [54]. Saponin disrupts the outer phospholipidic membrane carrying the structural lipopolysaccharide components of Gram positive bacterial (*S. aureus*) cell wall; leading to permeability of lipophilic solutes rendering the cell wall inactive [55]. In contrast, porins that constitute a selective barrier to the hydrophilic solutes are hindered, causing loss of osmotic activity of the cell membrane, resulting in bacterial cell death [55]. Action of tannins to inactivate microbial adhesions, enzyme synthesis (usually coagulase, staphylokinase and exoenzymes), cell envelope transport proteins and mineral uptake provide effective inhibitory activity [56]. Significant level of alkaloids provides promising anti-staphylococci activity due to its ability to inhibit the extra-outer membrane, that acts as a barrier for other compound(s) to diffuse into the bacterial cytosol and act similarly as DNA intercalating agents or topoisomerase inhibitors which adversely affect DNA replication and supercoiling [57].

Recorded antibacterial activities of the plant extracts against multi-antibiotic resistance *S. aureus* substantially corroborate the functional properties of the phytochemicals as a good source for effective lead compounds for anti-staphylococci drug candidates. Aqueous extract of these plants has shown high-level anti-staphylococci activities that could be harnessed to develop an antibacterial agent for SSTIs. Formulating these extracts with skin ointment, wound wash, antiseptics, and surgical wound dressing pad would accelerate effective healing process involving coagulation, epithelization, collagenation, wound contraction and reduce septic skin inflammation [58].

## Conclusion

The observed high occurrence of skin and soft tissue infections indicates increasing morbidity and continuous dissemination of biofilm-producing MDR-*S. aureus*. There is need to develop the significant phytochemical compounds such as alkaloids, phenolic, terpeniods and flavonoids in aqueous leave extracts of *Moringa oleifera, Acalypha wilkesiana, Azadirachta indica* and *Vernonia amygdalina* which are suitable potential substances for new anti-staphylococci agent. Further studies are recommended to effectively process and develop the extract into novel herbal formulations as skin therapeutic agents.

## Supplementary Information

**Additional file 1.**

## Data Availability

All data generated or analyzed during this study are included in this article and its supplementary information files.
