# Supplementary material for: Antibacterial activities of plant leaf extracts against multi-antibiotic resistant Staphylococcus aureus associated with skin and soft tissue infections
Source: BMC Complement Med Ther. 2022 Feb 21;22:47. doi: 10.1186/s12906-022-03527-y (PMC8862250; doi:10.1186/s12906-022-03527-y)
Supplement: Supplementary file 1 — Additional file 1. [file 12906_2022_3527_MOESM1_ESM.docx]

**Anti-staphylococci activities of plant leaf extracts against multi-antibiotic resistant *Staphylococcus aureus* associated with skin and soft tissue infections**

Akinduti P.A^1^, Emoh-Robinson V^1^, Obamoh-Triumphant H.F^1^, Obafemi Y.D^1^, Banjo T.T^2^

Supplementary 1 Table; Antimicrobial susceptibility pattern of the isolated Staphylococci strains

| code | AGE | SPECIMEN | TE | CAZ | CIP | CN | AMC | CRO | OFX | SXT | E | FOX | VA | LZD | MAR |
| --- | --- | --- | --- | --- | --- | --- | --- | --- | --- | --- | --- | --- | --- | --- | --- |
| S46 | 39 | wound | S | S | S | S | R | S | R | R | R | R | S | S | 0.25 |
| S59 | 50 | wound | R | S | S | R | R | R | R | R | S | R | R | S | 0.42 |
| S68 | 5 | ear | R | S | S | R | S | R | S | R | R | R | S | S | 0.42 |
| S38 | 20 | wound | R | R | R | R | R | R | R | R | S | R | R | R | 0.83 |
| S9 | 35 | pus | R | S | S | S | R | S | S | S | S | R | R | R | 0.42 |
| S22 | 6 | wound | R | R | S | S | S | R | S | R | S | R | S | S | 0.25 |
| S35 | 1 | abscess | R | R | R | R | R | R | R | R | R | R | R | R | 0.92 |
| S80 | 48 | abscess | R | R | S | R | S | S | R | R | R | R | R | S | 0.42 |
| S29 | 1 | ear | R | R | R | R | R | R | R | R | S | R | S | S | 0.67 |
| S28 | 21 | pus | R | R | R | R | R | R | R | R | R | R | R | R | 0.92 |
| S36 | 32 | abscess | S | R | R | R | S | R | R | R | S | R | S | S | 0.17 |
| S48 | 2 | ear | R | R | S | R | S | R | R | R | R | R | S | S | 0.42 |
| S57 | 25 | abscess | R | R | S | R | S | R | R | R | R | R | S | S | 0.42 |
| S77 | 62 | pus | R | R | S | S | S | R | S | R | S | R | S | S | 0.25 |
| S39 | 1 | ear | R | R | S | R | R | R | S | R | R | R | S | S | 0.5 |
| S41 | 72 | wound | R | R | R | R | R | R | R | R | S | R | S | S | 0.5 |
| S70 | 1 | ear | R | R | R | S | S | R | S | R | S | R | S | S | 0.25 |
| S73 | 7 | abscess | R | R | R | R | R | R | R | R | R | R | S | S | 0.67 |
| S5 | 22 | ear | R | S | S | S | S | S | S | R | S | R | R | S | 0.25 |
| S42 | 30 | wound | R | S | R | S | S | R | R | R | R | R | S | S | 0.25 |
| S6 | 41 | abscess | R | S | S | R | S | S | S | R | S | R | S | S | 0.25 |
| S58 | 9 | wound | S | R | S | S | R | R | S | R | S | R | S | S | 0.25 |
| S24 | 50 | ear | R | S | S | S | S | S | R | S | R | R | R | S | 0.17 |
| S10 | 25 | ear | R | R | R | R | S | R | R | R | R | R | R | R | 0.83 |
| S52 | 62 | wound | R | R | R | R | R | S | S | R | R | R | R | R | 0.58 |
| S63 | 3 | pus | R | R | S | R | R | S | S | R | R | R | R | S | 0.42 |
| S62 | 4 | wound | R | R | R | R | R | R | R | R | R | R | S | S | 0.83 |
| S75 | 13 | abscess | S | S | R | R | S | S | R | R | R | R | R | R | 0.33 |
| S16 | 9 | pus | R | R | R | R | R | R | R | R | R | R | R | R | 0.92 |
| S15 | 21 | wound | R | R | R | R | R | R | R | R | R | R | R | R | 0.75 |
| S4 | 17 | wound | R | R | R | R | R | R | R | R | R | R | R | R | 1 |
| S23 | 1 | pus | R | R | R | R | R | R | R | R | R | R | R | R | 0.83 |
| S79 | 32 | wound | R | R | R | S | R | R | R | R | R | R | R | R | 0.83 |
| S34 | 57 | wound | R | R | R | R | R | R | R | R | R | R | S | S | 0.83 |
| S54 | 49 | pus | R | R | R | R | R | R | R | R | R | R | R | R | 0.67 |
| S74 | 1 | ear | R | R | R | R | R | R | R | R | R | R | S | S | 0.67 |
| S78 | 74 | wound | R | S | R | R | R | S | R | R | R | R | S | S | 0.67 |
| S17 | 1 | pus | R | R | R | R | S | R | R | R | R | R | R | R | 0.67 |
| S82 | 23 | wound | R | R | R | S | R | R | R | R | R | R | S | S | 0.67 |
| S105 | 1 | ear | S | R | R | R | R | R | R | R | R | R | S | S | 0.5 |
| S11 | 52 | pus | R | R | R | R | R | R | R | R | R | R | S | S | 0.5 |
| S7 | 5 | ear | R | R | R | R | R | R | R | R | R | R | R | R | 0.75 |
| S89 | 2 | ear | R | S | R | R | S | R | R | R | R | R | S | S | 0.58 |
| S104 | 52 | ear | R | R | R | R | R | S | R | R | R | R | R | R | 0.67 |
| S95 | 1 | pus | R | R | R | R | R | R | R | R | R | R | S | S | 0.83 |
| S60 | 1 | ear | S | R | R | R | S | R | R | R | R | R | R | R | 0.58 |
| S66 | 5 | wound | R | R | R | S | S | S | R | R | R | R | S | S | 0.5 |
| S87 | 28 | abscess | R | R | R | R | R | R | R | R | R | R | S | S | 0.67 |
| S12 | 21 | pus | R | R | R | R | R | R | R | R | R | R | R | R | 0.67 |
| S114 | 1 | abscess | R | R | R | R | R | R | R | R | R | R | S | S | 0.58 |
| S71 | 67 | wound | R | R | R | R | R | R | R | R | R | R | S | S | 0.58 |
| S45 | 14 | wound | R | R | R | S | R | R | R | R | R | R | S | S | 0.67 |
| S103 | 21 | pus | R | R | R | R | R | R | S | R | R | R | S | R | 0.83 |
| S30 | 10 | ear | R | R | R | R | R | R | R | R | R | R | R | R | 1 |
| S92 | 48 | abscess | R | R | S | R | R | R | S | R | R | R | R | R | 0.75 |
| S100 | 21 | wound | R | R | R | R | R | R | R | R | R | R | R | R | 0.83 |
| S8 | 13 | ear | R | R | R | R | R | R | R | R | R | R | R | R | 1 |
| S90 | 1 | ear | R | R | R | R | R | R | R | R | R | R | R | R | 1 |
| S37 | 2 | wound | R | R | R | R | R | R | R | R | R | R | R | R | 1 |
| S49 | 50 | wound | R | R | R | R | R | R | R | R | R | R | R | R | 1 |
| S21 | 28 | abscess | R | R | R | R | R | R | R | R | R | R | R | R | 1 |
| S3 | 35 | wound | R | R | R | R | R | R | R | R | R | R | R | R | 1 |
| S6 | 49 | wound | R | R | R | R | R | R | R | R | R | R | R | R | 1 |
| S8 | 26 | wound | R | R | R | R | R | R | R | R | R | R | R | R | 1 |
| S13 | 23 | wound | R | R | R | R | R | R | R | R | R | R | R | R | 1 |
| S14 | 33 | wound | R | R | R | R | R | R | R | R | R | R | R | R | 1 |
